# A pilot study of the in vitro antimicrobial activity and in vivo residual activity of chlorhexidine and acetic acid/boric acid impregnated cleansing wipes

**DOI:** 10.1186/s12917-019-2098-z

**Published:** 2019-10-30

**Authors:** Rebecca Rafferty, Victoria H. Robinson, Jennifer Harris, Sally A. Argyle, Tim J. Nuttall

**Affiliations:** 1 University of Edinburgh, Royal (Dick) School of Veterinary Studies, Easter Bush Campus, Roslin, EH25 9RG UK; 2 The Dermatology Referral Service, 528 Paisley Road West, Glasgow, G51 1RN UK

**Keywords:** Topical, Antimicrobial wipe, Chlorhexidine, Acetic acid, Boric acid, Microbial infection, Cutaneous, Dog

## Abstract

**Background:**

Topical antimicrobials are recommended for first line treatment of surface and superficial infections in dogs. This is especially important given the increasing prevalence of antimicrobial resistant infections. Antimicrobial wipes have become popular, but there are a lack of controlled studies assessing their in vitro antimicrobial and in vivo residual activity. We aimed to assess the antimicrobial efficacy of two commercial antimicrobial wipes against frequently isolated pathogens.

Ten clinical and one reference isolate each of meticillin-susceptible *Staphylococcus pseudintermedius* (MSSP), meticillin-resistant *S. pseudintermedius* (MRSP), *Escherichia coli* (EC), extended spectrum beta-lactamase (ESBL) producing *E. coli* (ESBL-EC), *Pseudomonas aeruginosa* (PA) and *Malassezia pachydermatis* (MP) were tested using a modified Kirby-Bauer technique. Each isolate was tested against 6 mm discs of chlorhexidine (CHX) and acetic acid/boric acid (AABA) wipes, and positive and negative controls either overnight (bacteria) or for 3 days (*Malassezia*).

Healthy dogs were treated with the wipes and distilled water on a randomised flank (*n* = 5 each). Hair samples (1 cm; 0.1 g) taken at days 0, 1 and 3 were inoculated with an isolate of each organism. Zones of inhibition (ZI) were measured.

**Results:**

All isolates produced confluent growth with AABA and control wipes, except for the cleansing wipes and MP (median ZI 12 mm; 95% CI 8.2–15.8). The median (95% CI) CHX wipe ZIs (mm) were: MP 48.0 (47.0–49.0), MSSP 15.6 (14.2–17.0), MRSP 14.0 (13.6–14.4), EC 13.6 (12.0–15.2) and ESBL-EC 10.0 (9.4–10.6). PA showed confluent growth. The differences between the bacterial isolates was significant (Kruskal-Wallis *p* < 0.0001; post-tests MSSP = MRSP = EC > EBSL-EC > PA). Confluent growth was visible with all the hair samples.

**Conclusion:**

CHX but not AABA showed in vitro efficacy against MSSP, MRSP, EC and MP. ESBL-EC were less susceptible and there was no activity against PA. There was no residual activity on hair. Additional studies are required to determine efficacy of these products in clinically affected patients.

## Background

Antimicrobial resistance (AMR) is a growing concern in human and veterinary healthcare. Treatment with systemic antibiotics, particularly broad-spectrum agents, is an important driver for the selection and dissemination of AMR. There is therefore renewed interest in using effective antiseptics to reduce systemic antibiotic use. For example, topical antiseptic therapy, particularly with chlorhexidine shampoos, is recommended as first line therapy for surface and superficial pyodermas in dogs [1, 2].

Antiseptic wipes are popular for their ease of use compared to shampoos [3], but it is important to know that the products are effective. Chlorhexidine (CHX) containing wipes (CLX® wipes containing chlorhexidine, climbazole and trisEDTA; ICF, Cremona, Italy) and acetic acid/boric acid (AABA) wipes (Malacetic® wipes containing acetic and boric acid; Dechra® Veterinary Products, Shrewsbury, UK) are both marketed as antiseptic, antibacterial and/or antifungal cleansing wipes. One study evaluated the in vivo and in vitro activity of CHX wipes against *Malassezia pachydermatis* in an experimental model with 5 shar pei dogs [4]. Applying the wipes 1 or 2 times daily to the skin for 30 s significantly reduced *Malassezia pachydermatis* counts on contact plates. In vitro assays using the wipe solution demonstrated complete kill in 6 isolates after 15 min contact time. However, antibacterial efficacy was not assessed and there were no control groups in the study. To our knowledge, there are no other peer-reviewed publication on the efficacy of these antiseptic wipes.

The aim of this pilot study was to assess the in vitro antimicrobial efficacy and residual in vivo antimicrobial activity of these wipes against meticillin-susceptible *Staphylococcus pseudintermedius* (MSSP), meticillin-resistant *S. pseudintermedius* (MRSP), *Escherichia coli* (EC), extended spectrum beta-lactamase (ESBL) producing *E. coli* (ESBL-EC), *Pseudomonas aeruginosa* (PA) and *Malassezia pachydermatis* (MP) isolates. *S. pseudintermedius* and MP are commensals of canine skin and are frequently associated with microbial infections. PA, EC and ESBL-EC are less commonly isolated from cutaneous infections but they are important causes of ear, wound and surgical site infections [5–10].

## Results

The positive control antimicrobial discs produced the expected ZIs (zones of inhibition) for susceptibility based on the breakpoints observed in their clinical identification and antimicrobial susceptibility testing. Colony morphology and cytology was consistent with pure cultures. There was no growth on any of the negative control plates (i.e. without an inoculum).

### Antimicrobial activity in vitro

The CHX wipes produced significantly greater ZIs than the AABA wipes, paper towel or cleansing wipes for the MSSP, MRSP, EC, ESBL-EC and MP isolates (all *p* < 0.0001; see Table 1) but there was no difference between the AABA wipes, paper towel and cleansing wipes where most isolates achieved confluent growth (*p* = 0.05 to 1.0). All of the *PA* isolates achieved confluent growth with the CHX and AABA wipes, paper towel and cleansing wipes. There was a significant difference between the ZIs for the CHX wipes among the different bacteria with MSSP = MRSP = EC > ESBL-EC > PA (*p* < 0.0001). The ZIs for the reference isolates all fell within the ranges seen with the clinical isolates.

### Residual antimicrobial activity in vivo

All the bacterial and *Malassezia* isolates achieved confluent growth around all the hair samples at days 0, 1 and 3.

## Discussion

This study shows that the CHX wipes show in vitro efficacy against a range of pathogenic isolates relevant to skin and wound infections in animals, including MSSP, MRSP, EC, ESBL-EC and MP. The small differences between ZIs for MSSP, MRSP and EC were non-significant, but the ESBL-EC showed significantly smaller ZIs and all the PA isolates achieved confluent growth. In contrast, all of the isolates achieved confluent growth when incubated with the AABA impregnated wipes. Neither wipe demonstrated any persistent activity on hair samples after thorough application in five dogs.

Chlorhexidine is an effective broad-spectrum antimicrobial [11–14]. Chlorhexidine containing shampoos, ear cleaners and sprays have shown in vivo and in vitro efficacy against a range of organisms including *Pseudomonas* [15–21]. The reason for the poor efficacy against PA (and to some extent ESBL-EC) in this study is unknown. Resistance to chlorhexidine has been associated with antibiotic resistance, which may be relevant to the multi-drug resistance seen in the PA and ESBL-EC isolates used in this study [22]. Kandry and colleagues showed that 22 out 36 MDR PA isolates carried class I integrons with reduced susceptibility to biocides including chlorhexidine. Furthermore, quaternary ammonium compound resistance E (qacE) genes, which are associated with chlorhexidine resistance, were identified in 11 isolates [22]. Nevertheless, while treatment failures are seen, these have not been proven to be associated with resistance to chlorhexidine in staphylococci and other bacteria in veterinary medicine. The relationship between resistance genes to clinical failure or success is therefore unclear.

Uri and others (2016) reported that the minimal inhibitory concentration (MIC) of MDR (multiple drug resistant) PA was 0.94 g/L compared to the recommended concentration of 0.5 g/L. [23] The wipes in our study contain 0.3% (3 g/L) chlorhexidine, which is lower than that in many topical products (typically 2–4%; 20-40 g/L). Nevertheless, in vitro inhibition has been demonstrated with a 0.15% chlorhexidine ear cleaner, and chlorhexidine has MIC and minimal bactericidal concentrations (MBCs) of 0.6–10 mg/L against the ESBL-EC and 5–10 mg/L against the PA isolates used in this study (data submitted for publication). MBCs of chlorhexidine to PA in previous veterinary studies have been found to be between 5 and 28 mg/ml [24, 25] and MIC in human studies of 4 mg/ml [26]. which compares favourably to our submitted study. MICs for chlorhexidine against ESBL-EC in human clinical isolates was determined as < 1-2 mg/L [26, 27] and in avian isolates as 0.5-1 mg/L. [27] MBCs of ESBL-EC were 7.32 mg/L (3 min incubation) to 1.83 mg/L (10 min incubation) with a 4% chlorhexidine product, and 468.75 mg/L (3 and 10 min incubations) with a 3% chlorhexidine/0.5% climbazole product [23]. These additional studies have determined MIC/MBC comparable to the our submitted study.

Nonetheless, it is difficult to extrapolate the effective in vitro and in vivo concentration that bacteria would be exposed to using chlorhexidine-impregnated wipes. It is unknown how diffusion of the chlorhexidine, acetic acid/boric acid, and other ingredients into the surrounding MH and SD agar affected the concentration that the organisms were exposed to. However, studies of the antimicrobial efficacy of products with these ingredients in agar well diffusion [28, 29] and agar plated hair models [30, 31] suggest that they freely diffuse into the media.

The CHX wipes also contained trisEDTA, zinc gluconate, climbazole and a range of other ingredients (Table 2). Their antimicrobial efficacy and/or effect on the efficacy of the chlorhexidine is unknown. TrisEDTA exhibits very little antimicrobial efficacy by itself, although it can potentiate the activity of chlorhexidine with 21% of isolates [13] particularly at high concentrations (at least 25–50 g/L of a 4.2:1 combination TrisEDTA:chlorhexidine; data submitted for publication). It is unknown whether climbazole or chlorhexidine show additive or synergistic activity against MP, where the ZIs around the CHX disc were greater than the control antifungal disc. Fractional inhibition indices studies could be performed in future to determine if there is an additive or synergistic interaction between these compounds. Miconazole exhibits additive [13, 14] and synergistic [14] activity with chlorhexidine against some MSSP and MRSP isolates but is not known if this occurs with climbazole.

**Table 1:** Zones of inhibition (ZIs) of tested wipes. In vitro antimicrobial efficacy showing median (95% CI) of the zones of inhibition (ZIs) (mm; Con = confluent growth) of the chlorhexidine (CHX) wipes, acetic acid/boric acid (AABA) wipes, paper towel and cleansing wipes (MSSP = meticillin-susceptible *S. pseudintermedius*; MRSP = meticillin-resistant *S. pseudintermedius*; EC = *E. coli*; ESBL-EC = extended spectrum beta-lactamase producing *E. coli*; PA = *Pseudomonas aeruginosa*; MP = *Malassezia pachydermatis*; *n* = 11 [10 clinical isolates and 1 Public Health England National Collection of Types Culture (NCTC) and Public Health England National Collection of Pathogenic Fungi (NCPF reference isolates])

	MP	MSSP	MRSP	EC	ESBL-EC	PA
CHX wipes	48.0 (47.0-49.0	15.6 (14.2-17.0)	14.0 (13.6-14.4)	13.6 (12.0-15.2)	10.0 (9.4-10.6)	Con
AABA wipes	Con	Con	Con	Con	Con	Con
Cleansing wipes	12 (8.2-15.8)	Con	Con	Con	Con	Con
Paper towel	Con	Con	Con	Con	Con	Con

**Table 2:** Composition of the antimicrobial and control wipes according to the manufacturer’s data

Chlorhexidine (CHX) wipes (CLX®wipes, ICF®, Cremona, Italy)	0.3% Chlorhexidine 0.5% Climbazole 1% Zinc gluconate TrisEDTA Glycerin Non-ionic surfactant Benzyl alcohol Propylene glycol Perfume Demineralized water
Acetic acid/boric acid (AABA) wipes (Malacetic® wipes (Dechra® Veterinary Products, Shrewsbury, UK)	2% Acetic acid 2% Boric acid Propylene glycol Glycerin Fragrance
Simple® Kind to Skin Facial Cleansing Wipes (Unilever, Leatherhead, UK)	Aqua Benzoic acid Cetearyl isononanoate Ceteareth-12 Ceteareth-20 Cetearyl alcohol Citric acid Dehydroacetic acid Disodium EDTA Glycerin Glyceryl stearate Panthenol Pantolactone Phenoxyethanol Sodium citrate Tocopheryl acetate

No antimicrobial activity was seen with the AABA wipes. Similar findings have been reported for 2% acetic acid/2% boric acid ear cleaners and shampoos in broth dilution studies [20, 21].Several human studies have looked at the antibacterial activity of acetic acid. MICs for PA and EC varied from 1.25–3.1 mg/ml [32–34] and 3.1 mg/ml for ESBL-EC [34]. This suggests that the antimicrobial activity of acetic acid varies within and between studies. There are few studies assessing boric acid, but reported MICs are 7.6 mg/ml against single isolates of EC and PA [35] and 0.385–0.77 mg/ml for two PA isolates [36] thus showing variation. Therefore, although acetic acid and boric acid are antimicrobial, dilution and/or interaction with other ingredients in the wipes may reduce the antimicrobial efficacy.

Our study also shows that there was no residual in vitro activity from hairs treated with these antiseptic wipes against the tested isolates. Only one isolate of each organism was tested, but we selected the isolate with the largest ZI to the CHX wipes to best show antimicrobial activity. The same isolates were used for AABA wipes as the earlier confluent growth prevented selection of an isolate in the same way. It is possible that there may have been some short-term activity as the first samples were collected 24 h after application. This study could have been improved by collecting a hair sample at shorter time intervals such as 30mins. The authors are unaware of any short term clinical studies using this product. However, studies with identical methodology have shown that canine hairs show in vitro antimicrobial activity for up to 10 days after application of chlorhexidine containing shampoos and rinses [30, 31]. The reasons for the lack of persistent activity of the CHX wipes is unclear, but may relate to the low concentration of chlorhexidine in the formulation and further studies are indicated.

This study only used 10 clinical and one reference isolate of each organism for the in vitro experiment. Unfortunately, a reference isolate for MRSP was not available from NCTC at the time of this study. Ideally, larger numbers of clinical isolates would have been studied. However, the clinical isolates were chosen at random across different time periods to maximize representative sampling and avoid selection bias. The lack of bias is also supported by the narrow range of the ZIs (most of the 95% CI differed only 10% from the median results). Furthermore, the results for the reference isolates sat within the ranges established for the clinical isolates. Wide variability in the results would have suggested that much larger numbers of isolates would have been required to accurately demonstrate the relative in vitro antimicrobial activity of these products.

The study design was chosen to reflect the activity of the wipes rather than their ingredients, which have been established and would be less clinically relevant to the products as used. The experimental model was used to provide data on comparative in vitro antimicrobial activity. However, it is important to note that the model does not represent an in vitro disc diffusion test of the wipes and the results must not be read as breakpoints implying clinical susceptibility or resistance.

While these results demonstrate the in vitro antimicrobial activity of the CHX, further studies are required to demonstrate clinical efficacy. This cannot be assumed, as, for example, Boonyasiri and colleagues [37] showed that there was no benefit to using 2% chlorhexidine impregnated wash cloths versus non-antimicrobial soap cloths for cleaning patients in a human ICU ward. The median time to MDR bacterial colonisation was 5 days with no significant difference in hospitalisation time or incidence of hospital acquired infections (including ESBL-EC, Meticillin –resistant *Staphylococcus aureus*, MDR *Klebsiella pneumoniae* and *Acinetobacter baumanii)* [37]. Recent meta-analysis by Patel and colleagues have found that there is no beneficial effect of daily use of chlorhexidine containing products to prevent gram negative bacterial infections caused by EC, PA, *Acinetobacter, Klebsiella* and *Enterobacter* [38].

## Conclusion

This study has shown that CHX wipes inhibited the in vitro growth of a range of pathogens relevant to skin and wound infections in animals. AABA wipes were ineffective in vitro. Growth of the MSSP*,* MRSP, EC and MP isolates were inhibited, but the ESBL *E. coli* isolates appeared to be less susceptible and all the PA isolates achieved confluent growth. Clinicians should therefore use clinical signs, cytology, and where necessary culture, to determine whether treatment of an infection with the CHX product is appropriate. Finally, there was no residual activity on the hair suggesting that these CHX wipes need to be used at least once a day on active infections. Further studies are, however, required to establish whether these results can be replicated with larger samples and to demonstrate in vivo clinical efficacy.

## Methods

### Microbial isolates

Ten isolates each of MRSP, MSSP, EC, ESBL-EC, PA and MP were obtained from cases of canine otitis, pyoderma and wound infections. These isolates were randomly selected from samples submitted to the University of Edinburgh Veterinary Pathology Unit microbiology laboratory for routine microbial culture and susceptibility testing. Random numbers were computer generated to select samples using the laboratory submission numbers. Samples with insufficient material, growth characteristics and/or data were disregarded and the random selection process repeated until the target number was obtained. The organisms were speciated and their antimicrobial susceptibility established using standard methods employed by this accredited laboratory using CLSI guidelines [39, 40]. Reference isolates were obtained from the Public Health England National Collection of Type Cultures (NCTC) and Public Health England National Collection of Pathogenic Fungi (NCPF). These were *Staphylococcus pseudintermedius* NCTC 7151, *E. coli* NCTC 12241, CTX-M-15 ESBL *E. coli* NCTC 13353, *Pseudomonas aeruginosa* NCTC 10662 and *Malassezia pachydermatis* NCPF 3667. *S. aureus* EMRSA-15 NCTC 13142 was used in the absence of an MRSP reference isolate. All the isolates were stored in tryptone soya broth (bacteria) or Sabouraud dextrose broth (*Malassezia*) with 15% glycerine at -80 °C until required. Isolates were defrosted and cultured overnight on Columbia 5% horse blood agar (bacteria) at 37 °C or for three days on Sabouraud dextrose agar (*Malassezia*) at 37 °C with 5% CO_2_. Colonies were washed and diluted in sterile phosphate buffered saline (PBS) to a visually assessed 0.5 McFarland standard. All the media were obtained from Oxoid™ (ThermoFisher Scientific™, Basingstoke, UK).

### Agar diffusion studies

The microbial isolates were spread onto Mueller-Hinton (MH; bacteria) and Sabouraud dextrose (SD; *Malassezia*) agar plates in a standard manner to achieve confluent growth. 6 mm discs were cut using sterile instruments from the chlorhexidine (CHX) and acetic acid/boric acid (AABA) impregnated wipes, and negative controls (sterile autoclaved Simple® Kind to Skin Facial Cleansing Wipes [Unilever, Leatherhead, UK] and paper towel). See Table 2 for further details of the composition of the wipes. One disc of each was added to each plate. Antimicrobial impregnated discs to which the isolate had previously demonstrated susceptibility were used as positive controls. The choice of antimicrobial was based on previous culture and susceptibility testing performed to accepted CLSI guidelines [39, 40]. These included: 30 μg cephalexin, 30 μg doxycycline, 10 μg gentamicin, 30 μg amoxicillin/clavulanic acid, 30 μg ceftazidime, 30 μg amikacin, 5 μg enrofloxacin and 25 μg fluconazole (Oxoid™ ThermoFisher Scientific™, Basingstoke, UK). All the plates were set up in duplicate. Negative control MH and SD agar plates with the test, positive and negative control discs but without microbial isolates, were also included. Each isolate was incubated overnight (bacteria) or for 3 days (*Malassezia*) at 37 °C and 5% CO_2_. The plates were then examined for microbial growth and the zones of inhibition (ZIs) were measured to the edge of the microbial growth. Colony morphology and cytological examination were used to determine the purity of the microbial growth.

### In vivo residual activity study

Ethical approval through the parent institute was granted for this part of the study and all owners gave informed written consent. To evaluate the in vivo duration of activity, an area of haired skin on the lateral thorax in healthy dogs was vigorously rubbed with the CHX (*n* = 5) or AABA (n = 5) wipes until the hair and underlying skin was thoroughly soaked. The other side of each dog was similarly treated with sterile distilled water. Treatment allocation to CHX or AABA and to the right or left side was randomly assigned using a coin toss with the investigator blinded to the allocation. No further topical treatment or grooming was permitted during the study period and the dogs were kept dry. Hair samples were collected from the treated sites at day 0 (i.e. before treatment), and then on days 1 and 3. The hair was cut and weighed so that each sample was the same size (1 cm length) and weight (0.1 g). Plates with an MSSP, MRSP, EC, ESBL-EC and MP isolate shown to be most susceptible to the CHX wipes were prepared as above. The PA isolate was selected at random as the isolates had all achieved confluent growth in the first part of the study. Each plate was then inoculated with the hair samples and positive control antimicrobial disc, and incubated as above. The ZI around each hair sample was examined and measured across the midpoint of the sample. Colony morphology and cytological examination were used to determine the purity of the microbial growth.

### Data analysis

The ZIs were recorded in mm. The median and 95% confidence intervals (CIs) were calculated for the 11 isolates of each organism. Kruskal-Wallis tests with post-test analyses was used to compare the ZIs between the test and control wipes for each microbial isolate (i.e. CHX versus AABA for MSSP, MRSP, EC, ESBL-EC, PA and MP) and then between the ZIs for each wipe among the bacterial isolates (i.e. MSSP versus MRSP, EC, ESBL-EC and PA for CHX and AABA). The MP isolates were not included in the latter analysis as these used a different culture methodology.

## Data Availability

The datasets used and analysed during the current study are available from the corresponding authors on reasonable request.

## Abbreviations

AABA: Acetic acid/boric acid
AMR: Antimicrobial resistance
CHX: Chlorhexidine
EC: *Escherichia coli*
ESBL-EC: Extended spectrum beta-lactamase (ESBL) producing *E. coli*
MBC: Minimal bactericidal concentrations
MDR: Multiple drug resistance
MIC: Minimal inhibitory concentration
MP: *Malassezia pachydermatis*
MRSP: Meticillin-resistant *Staphylococcus. pseudintermedius*
MSSP: Meticillin-susceptible *Staphylococcus pseudintermedius*
NCPF: Public Health England National Collection of Pathogenic Fungi
NCTC: Public Health England National Collection of Types Culture
PA: *Pseudomonas aeruginosa*
qacE: Quaternary ammonium compound resistance E
ZI: Zones of inhibition
